# P-1587. The Utility of Using FilmArray Meningitis-Encephalitis PCR Panel (MEP) in Children Admitted with Meningitis and Encephalitis at LUMC. Is it a Useful Diagnostic Stewardship Tool?

**DOI:** 10.1093/ofid/ofae631.1754

**Published:** 2025-01-29

**Authors:** Ban H AL-Sayyed, Kelli Covington, Pamela Nicoski, Marina Feffer, Madeleine Heyn, Gabriella Hunter-McElroy, Shibangi Pal, Louise Lie, Jenin Mannaa

**Affiliations:** Loyola University Hospital and Medical Center, Maywood, Illinois; Loyola University Hospital and Medical Center, Maywood, Illinois; Loyola University Hospital and Medical Center, Maywood, Illinois; Loyola University Chicago, Maywood, Illinois; Stritch School of Medicine, Maywood, Illinois; Stritch School of Medicine, Maywood, Illinois; Stritch School of Medicine, Maywood, Illinois; Loyola University Medical Center, Maywood, Illinois; Nova Southeastern University, Fort Lauderdale, Florida

## Abstract

**Background:**

MEP allows for rapid detection or exclusion of pathogens causing meningitis and encephalitis (ME) in neonates and children. Studies showed its high sensitivity and specificity as a tool in diagnosing ME and reducing duration of antibiotics. We conducted a retrospective study in our institution to evaluate MEP usage in our pediatric population suspected to have ME, assess the agreement between MEP and CSF culture and cell count results, and measure the impact of MEP results on discontinuing empiric antimicrobials.

**Figure:**
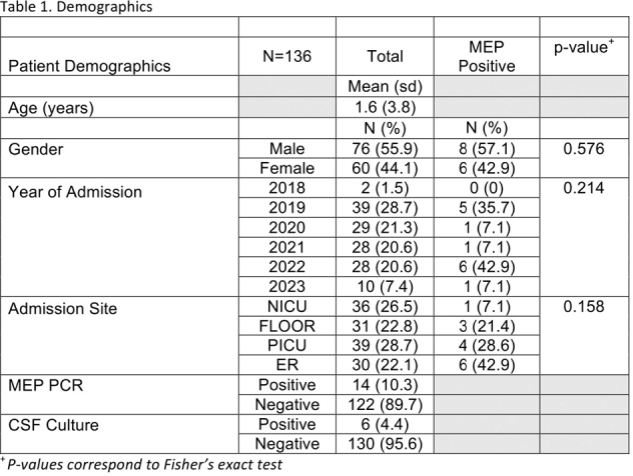

**Methods:**

We reviewed electronic records of patients aged 0–18 years admitted with possible ME from January 1, 2019 to April 30, 2023. We excluded patients with VP shunt infections and malfunctions, immunocompromised hosts, patients who did not receive antibiotics, and cases who had CSF culture done without MEP on admission.

**Figure:**
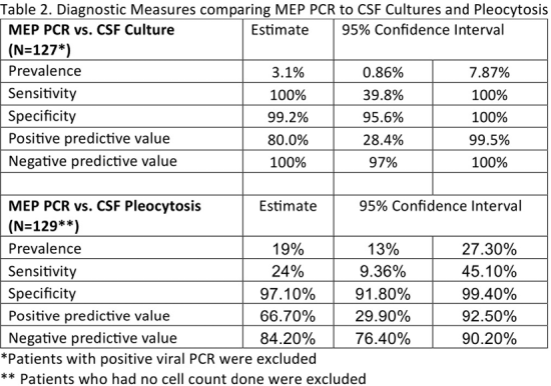

**Results:**

We included 136 patients admitted with possible ME and treated with antibiotics. Of all patients, 6.5% had viral PCR detected (n=9), 3.7% had bacterial PCR detected (n=5), 89% had negative PCR (n=122). Antibiotics were started before lumbar puncture in 77% of cases (n= 105). Only 23% were tapped before initiation of antibiotics (n = 31). There was 99.2% positive agreement between MEP and CSF culture results after viral isolates were removed (n=126/127). CSF pleocytosis was present in 19.4% of cases (n=25/129), and results were discordant with MEP in 17.1% of cases (n=22/129). Empiric vancomycin was started in 37% of patients who had no additional focus of infection on admission (n=41/111), and was discontinued within 12 hours of MEP results in 51% of cases (n=21/41). Empiric antibiotics were discontinued within 36 hours of negative MEP results in 31.5% of cases (n=35/111). Acyclovir was combined with antibiotics empirically in 13.5% of cases only (n=15/111).

**Figure:**
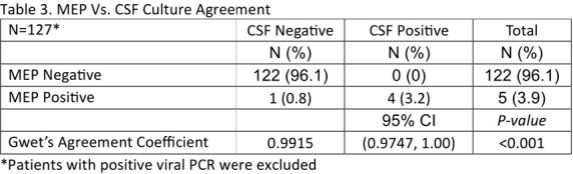

**Conclusion:**

MEP is an important diagnostic stewardship tool. It is highly sensitive and specific in diagnosing ME in children. However, agreement with CSF cultures might be limited by antibiotic treatment prior to lumbar puncture. MEP helps in quick de-escalation of empiric antibiotics and reduces unnecessary prolonged therapy in children treated with antibiotics before obtaining CSF. Using CSF pleocytosis as an indication for MEP testing has a low yield.

**Figure:**
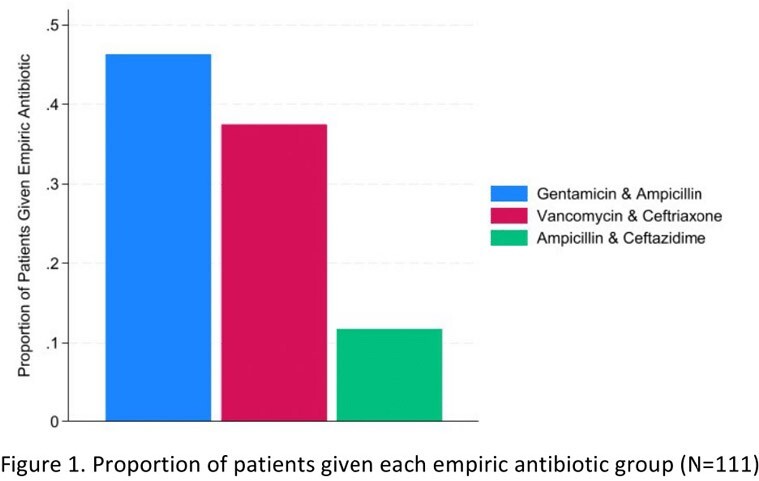

**Disclosures:**

**All Authors**: No reported disclosures

